# Pubertal Dynamics of Sertoli and Leydig Cell Dysfunction in Klinefelter Syndrome

**DOI:** 10.1111/cen.70159

**Published:** 2026-06-11

**Authors:** Tredez Axelle, Christine Lefevre, Simon Boussion, Cindy Colson, Iva Gueorguieva, Francois Marcelli, Anne Laure Barbotin, Pascal Pigny, Hamza Benderradji, Clara Leroy

**Affiliations:** ^1^ Department of Pediatric Endocrinology, DevGen, Reference Centre for Genital Development Abnormalities, CHU Lille University of Lille Lille France; ^2^ Department of Genetic, DevGen, Reference Centre for Genital Development Abnormalities, CHU Lille University of Lille Lille France; ^3^ Department of Andrology, Urology and Renal Transplantation University of Lille, CHU Lille Lille France; ^4^ Department of Reproductive Biology‐Spermiology‐CECOS University of Lille, CHU Lille Lille France; ^5^ University of Lille, CHU‐Lille, Inserm U1172, Lille Neuroscience & Cognition Lille France; ^6^ Department of Biochemistry and Molecular Biology, Hormonology, Metabolism–Nutrition and Oncology Unit, CHU‐Lille University of Lille Lille France; ^7^ U1277‐CANTHER‐Cancer‐Heterogeneity Plasticity and Resistance to Therapies, Inserm, CHU Lille University of Lille Lille France; ^8^ Department of Endocrinology, Diabetology, and Metabolism University of Lille, CHU Lille Lille France

**Keywords:** Klinefelter syndrome, puberty, Sertoli and Leydig cell dysfunction

## Abstract

**Context:**

Klinefelter syndrome (KS), defined by a 47, XXY karyotype, is commonly associated with progressive testicular failure. The precise timing of Sertoli and Leydig cell dysfunction during puberty remains unclear.

**Objective:**

To determine the onset and progression of testicular insufficiency during puberty in KS, and to assess whether diagnostic timing (prenatal vs postnatal) impacts pubertal phenotype.

**Methods:**

We conducted a retrospective, single‐centre study of 57 patients with KS aged 9–25 years, followed from the prepubertal period at Lille University Hospital. Longitudinal clinical and hormonal data were analysed according to pubertal stage rather than chronological age.

**Results:**

All patients entered puberty spontaneously at a physiological age (mean 12.6 years), but experienced a slow pubertal tempo (mean duration: 4.08 years). Sertoli cell dysfunction appeared within 12 months of pubertal onset, with rising FSH and declining AMH and inhibin B levels. Inhibin B concentrations never reached the reference range for Tanner stage 5 and declined below the adult threshold (92 pg/mL) 2 years after pubertal onset. Leydig cell dysfunction, defined by elevated LH and low‐normal testosterone, emerged after 36 months. At puberty completion, 94% had Sertoli cell insufficiency (i.e. FSH level > 7.19 IU/L) and 80% had Leydig cell insufficiency (LH > 5.88 IU/L). Postnatally diagnosed patients exhibited significantly higher LH (*p* = 0.03) and lower inhibin B (*p* = 0.01) levels.

**Conclusion:**

Testicular insufficiency in KS begins approximately 18 months after pubertal onset, with Sertoli cell failure preceding Leydig cell decline. Early diagnosis and longitudinal monitoring are essential to guide endocrine management and to support individualized counselling regarding fertility preservation strategies, which should be considered according to pubertal stage rather than chronological age.

## 1 Introduction

Klinefelter syndrome (KS) is the most common sex chromosome disorder in males, affecting approximately 1 in 500 to 1 in 800 boys [1]. It is characterised by the presence of one or more additional X chromosomes in individuals with a typical male 46, XY karyotype. The extra X chromosome may be found in all cells or only in a proportion of them (mosaicism). The classical form, with a 47, XXY karyotype, accounts for 80%–90% of all KS cases [2].

Despite its prevalence, KS remains significantly underdiagnosed: only about 25% of affected males receive a diagnosis during their lifetime [3], and fewer than 10% are identified before puberty. A minority of boys are diagnosed prenatally, typically due to suspicion of chromosomal abnormalities unrelated to KS [4, 5]. In neonates with KS, initial signs may include microphallus and cryptorchidism [5, 6]. During adolescence, gynaecomastia is common but usually transient [6, 7]. Testicular volume is reduced from childhood, and during puberty, testes fail to undergo normal growth, resulting in small, firm testes in adulthood [6, 8]. Histologically, the testes show progressive fibrosis and hyalinization of the interstitial and peritubular tissues, accompanied by reactive Leydig cell hyperplasia [9], a process that accelerates during puberty [10].

Monitoring testicular function is a key aspect of long‐term care in KS patients [11]. KS also represents the most frequent genetic cause of male infertility, found in approximately 11% of azoospermic men [12, 13]. Up to 80% of individuals with KS experience hypogonadism by adulthood [1]. However, the precise timing of testicular failure during adolescence in boys with KS remains unclear. While spontaneous onset of puberty is generally observed, the tempo of pubertal progression and the onset of testicular insufficiency are poorly characterised. Moreover, there is currently no consensus or established recommendation regarding the evaluation of fertility potential in boys diagnosed with KS prior to puberty.

*The primary objective* of this study was to determine the timing of the onset of testicular insufficiency during puberty in boys with Klinefelter syndrome.

*Secondary objectives were*: (1) to characterise phenotypic features at the end of puberty in patients followed since childhood, with particular attention to Leydig cell dysfunction; and (2) to assess, based on clinical practice observations, whether the circumstances of diagnosis (prenatal vs. postnatal) could serve as a prognostic factor.

## 2 Patients and Methods

### 2.1 Study Design

A retrospective, observational and descriptive study was conducted at Lille University Hospital. Patients included were those with a confirmed diagnosis of Klinefelter syndrome and a documented medical follow‐up from childhood, aged between 9 and 25 years at the time of data collection (i.e. born between 1997 and 2013). The year 1997 was chosen as the lower limit because it marks the initiation of systematic follow‐up protocols for boys with Klinefelter syndrome at Lille University Hospital. Boys younger than 9 years at the time of data collection were excluded, as they had not yet reached the age relevant for pubertal onset. For the others data were collected every 6 months to follow the progression of puberty.

This study was conducted in accordance with the principles of the Declaration of Helsinki and was approved by the Ethics Committee of Lille University Hospital (No. DEC23‐1593). In compliance with French regulations, all patients received written information about the study, and their non‐opposition to the use of their medical data was duly obtained.

### 2.2 Inclusion and Exclusion Criteria

**Inclusion criteria:**

Patients were eligible if they met all of the following:

- Confirmed diagnosis of homogeneous, non‐mosaic Klinefelter syndrome by karyotype revealing a formula 47XXY in all cells,
- Regular medical follow‐up at Lille University Hospital,
- Age between 9 and 25 years at the time of data collection.

**Exclusion criteria:**

Patients were excluded if any of the following applied:

- Presence of additional genetic abnormalities,
- Phenotypic presentation not fully explained by Klinefelter syndrome, with ongoing genetic investigations.

## 3 Patients and Study Outcomes

### 3.1 Data Collection

Clinical, anthropometric, pubertal staging and hormonal data were extracted from electronic medical records. Pubertal staging was assessed according to Tanner stages, as recorded during clinical visits [14]. Laboratory values included serum levels of luteinizing hormone (LH), Follicle‐stimulating hormone (FSH), Anti‐Müllerian Hormone (AMH), inhibin B, sex hormone‐binding globulin (SHBG), total testosterone, free testosterone and bioavailable testosterone.

Pubertal onset was defined as Tanner stage 2, corresponding to a testicular volume of 4 mL at clinical examination. Auxological and biochemical data were collected every 6 months starting from the onset of puberty. The end of puberty was assessed by the end of the pubertal growth spurt.

Leydig cell dysfunction was defined by an elevated LH level, exceeding the upper limit of the reference range established by Konforte et al., (LH level > 5.88 IU/L) [15]. This dysfunction was considered compensated when total testosterone levels remained within the normal range for Tanner stage 5, defined as > 0.99 ng/mL (3.4 nmol/L).

Sertoli cell dysfunction was defined by an FSH level above the normal range for the corresponding Tanner stage, based on Konforte et al., (FSH level > 7.19 IU/L) [15]. AMH reference ranges by Tanner stage were defined according to Demirjian et al. [16].

Complete clinical and hormonal data were available for 57 patients with Klinefelter syndrome and were included in the final analysis. The study flowchart is presented in Figure 1.

**Figure 1:**
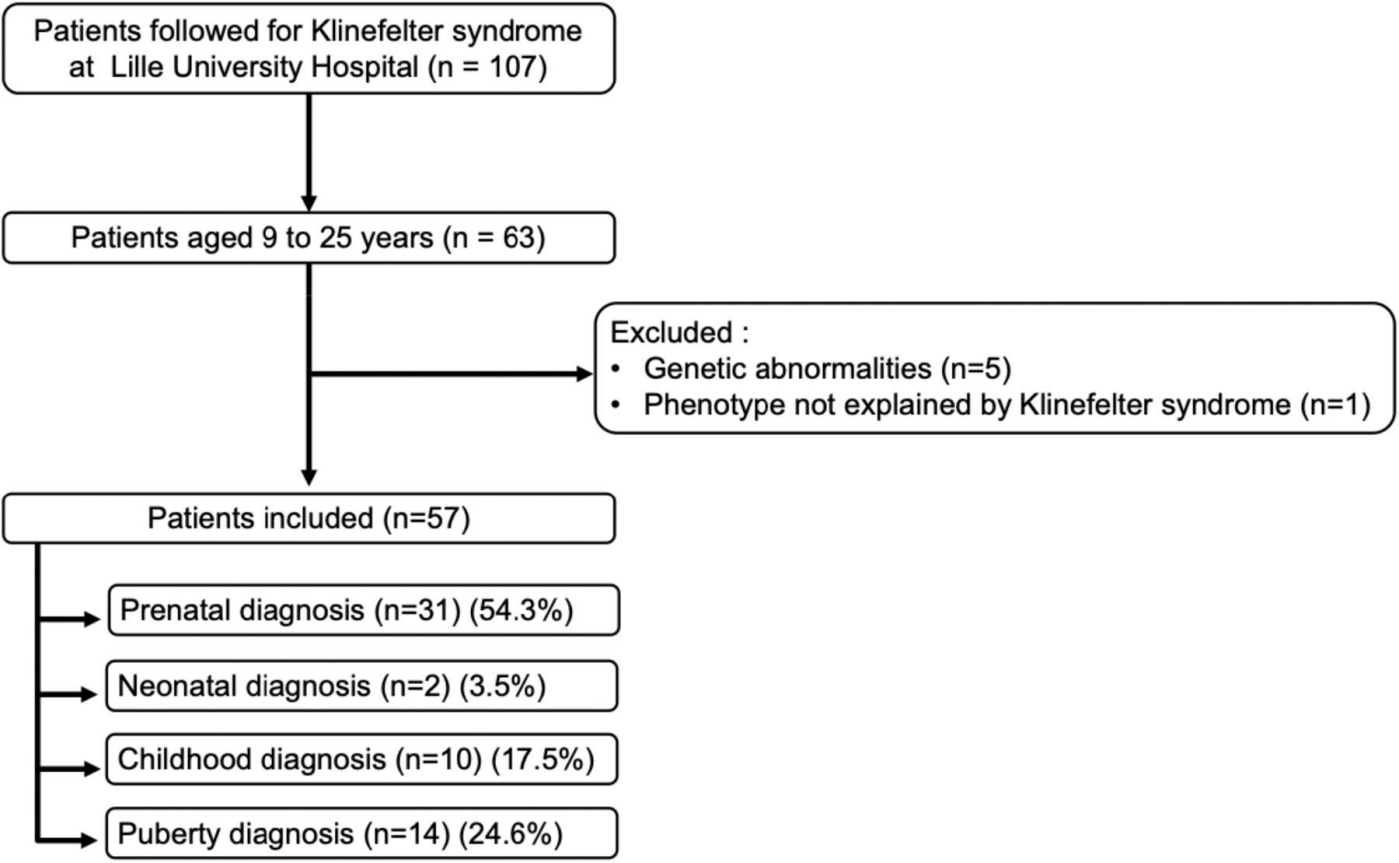
Flow chart of the study. A total of 57 out of 63 patients (aged 9–25 years) with Klinefelter syndrome were included in the study.

Testosterone replacement therapy (TRT) was initiated during follow‐up in the presence of clinical signs of androgen deficiency and biochemical hypogonadism, defined by low or low‐normal testosterone levels associated with elevated LH concentrations. TRT was prescribed according to routine clinical practice, using intramuscular testosterone esters with progressive dose escalation to achieve adult replacement doses by late puberty [5].

### 3.2 Hormone Assays

All hormone assays were performed in the same laboratory using serum samples collected at 9:00 a.m. to minimise circadian variability.

- **FSH, LH and total testosterone** were measured using immunoassays on an Architect analyzer (Abbott Laboratories, USA). Limits of quantification were 0.2 IU/L for FSH, 0.5 IU/L for LH and 0.04 ng/mL (0.14 nmol/L) for testosterone. Intra‐ and inter‐assay CVs ranged from 3.1% to 5.6% for FSH, 4.3% to 6.4% for LH and 4.7% to 5.2% for testosterone [17].
- **AMH** was measured using a fully automated sandwich chemiluminescent immunoassay (DXI Access, Beckman Coulter, USA), with a lower limit of quantification of 0.47 pmol/L. Values below this threshold were considered undetectable. Intra‐ and inter‐assay coefficients of variation (CVs) were < 2% and < 3%, respectively [18].
- **Inhibin B** was measured using the Inhibin B Gen II ELISA kit (Beckman Coulter, Villepinte, France), with a detection limit of 8 pg/mL. Values below this limit were considered undetectable [19].
- Serum concentrations of **SHBG** were measured by immunoradiometric assay (IRMA), using a commercial kit (Orion Dignostica, Espoo, Finland) and are referred to as iSHBG; the interassay CV is 8% a sandwich chemiluminescent immunoassay on a DXI Access analyzer (Beckman Coulter, USA). Intra‐ and inter‐assay coefficients of variation (CVs) were < 2% and < 3%, respectively.
- **Bioavailable testosterone and free testosterone** were calculated based on the Vermeulen equation that incorporate serum levels of total testosterone, albumin and SHBG.

### 3.3 Statistical Analysis

Qualitative variables were described as frequencies and percentages. Quantitative variables with a Gaussian distribution were expressed as mean ± standard deviation, whereas non‐Gaussian variables were reported as median and interquartile range (IQR). Normality of distribution was assessed using histograms and the Shapiro–Wilk test. For statistical purposes, when hormone serum levels were undetectable, they were assigned the value of the lower limit of quantification of the corresponding assay.

Comparisons between two groups were performed using Student's *t*‐test for normally distributed continuous variables, and the Mann–Whitney *U* test for non‐normally distributed continuous variables.

All statistical tests were two‐tailed, and a *p*‐value < 0.05 was considered statistically significant. Analyses were performed using GraphPad Prism, version 10.5.0 (GraphPad Software, La Jolla, CA, USA).

## 4 Results

### 4.1 Patients' Characteristics

Out of 63 patients aged 9 to 25 years with a confirmed diagnosis of Klinefelter syndrome, 57 were included in the final analysis. The study flowchart is presented in Figure 1.

Among the 57 included patients, 31 (54.4%) were diagnosed prenatally, primarily through routine genetic testing performed for unrelated indications. These included advanced maternal age in 18 cases (58%), increased risk of trisomy 21 on prenatal screening in 9 cases (29%), and a family history of chromosomal abnormalities in 2 cases (6%). In one case (3%), the diagnosis was prompted by the investigation of a congenital heart defect, and in another (3%) by the presence of polyhydramnios. These indications reflect clinical situations in which prenatal karyotyping is more likely to be performed, and therefore this subgroup may not be fully representative of the general population, but rather of a selected population undergoing genetic investigation.

The remaining 26 patients (45.6%) were diagnosed postnatally, either incidentally or in response to clinical features suggestive of Klinefelter syndrome. The reasons for karyotyping in this group included foetal growth restriction (1 case, 4%), bilateral cryptorchidism (1 case, 4%), speech and learning difficulties (7 cases, 27%), arrested pubertal progression (3 cases, 11%), small testicular volume (7 cases, 27%), gynaecomastia (4 cases, 15%), tall stature with disproportionately long legs (1 case, 4%), and obesity (2 cases, 8%).

The mean age at the time of data collection was 18.5  ±  4.18 years, and the mean age at the initiation of medical follow‐up was 9.27  ±  6.72 years.

Anthropometric, clinical and hormonal characteristics at the onset and at the end of puberty are summarised in Table 1.

**Table 1:** Description of the study population at the onset and completion of puberty.

	Onset of puberty (P2)	End of puberty (P5)
Number of individuals	57	36
Age (mean ± SD, years)	12.6 ± 1.26	17 ± 1.42
High (mean ± SD, cm)	159 ± 10.1	184 ± 6.788
BMI (mean ± SD, kg/m^2^)	18.1 ± 5.5	22.9 ± 7.22
Gynaecomastia (%)		42
HRT (%)		72
Age at HRT start (mean ± SD, years)		16.6 ± 1.59
Testosterone (median (IQR), ng/mL)	0.28 (0.16–1.63)	2.94 (2–4.55)
LH (mean ± SD, IU/L)	0.97 ± 0.81	13.41 ± 6.34
AMH (median (IQR), pmol/L)	712 (410.2–1005)	10.35 (7.4–26.45)
FSH (mean ± SD, IU/L)	2.90 ± 2.87	30.3 ± 14.41

*Note:* 36 patients had completed puberty at the time of analysis.

Abbreviations: HRT, hormone replacement therapy; IQR, Interquartile range (first and third quartiles); SD, standard deviation.

### 4.2 Characteristics at Pubertal Onset (Tanner Stage 2)

The mean age at the onset of puberty, defined as Tanner stage 2, was 12.6  ± 1.26 years. At that time, the mean height was 159  ± 10.1 cm, corresponding to approximately +0.5 standard deviation (SD) on the AFPA growth charts [20]. The mean body mass index (BMI) was 18.1 ±  5.5 kg/m², within the normal range according to the International Obesity Task Force (IOTF) reference curves [21].

At pubertal onset, the average serum LH level was 0.97 ± 0.81 IU/L (Table 1), below the upper reference threshold of 1.18 IU/L [15]. The median and the interquartile range of serum AMH level were 712 (410.2–1005) pmol/L, within the normal reference range [16]. Mean serum FSH level was 2.90 ± 2.87 IU/L, also within the expected range [15].

These data indicate that puberty began at a typical age in this cohort, with no biochemical evidence of Leydig or Sertoli cell dysfunction at the onset.

### 4.3 Characteristics at Completion of Puberty (Tanner Stage 5)

At the time of analysis, 36 patients (63.15%) had completed puberty. The mean duration of puberty was 4.08 ± 1.06 years, with an average age at completion of 17 ± 1.42 years.

Auxological parameters showed a mean final height of 184.1 ± 6.79 cm, corresponding to +1 SD on the AFPA growth charts. Mean BMI at the end of puberty was 22.9 ± 7.22 kg/m², falling within the overweight category according to IOTF standards [21].

Testicular volume remained significantly below the normal expected value of approximately 25 mL in healthy individuals. Mean volume was 3.95 ± 1.91 mL on the right and 4.0 ± 1.88 mL on the left.

Biochemically, the median and the interquartile range of serum testosterone level at the end of puberty were 2.94 ng/mL (IQR: 2.0–4.55), corresponding to 10.2 nmol/L (IQR: 6.9–15.7), within the expected range for Tanner stage 5 (0.87–7.59 ng/mL; 3.0–26.3 nmol/L) [15]. However, 29 of the 36 patients (80%) met criteria for Leydig cell insufficiency, defined as elevated LH with normal or low testosterone levels. Mean serum LH concentration was 13.5 ± 6.34 IU/L, exceeding the upper reference threshold (> 5.88 IU/L) [15].

Sertoli cell function was also severely impaired: the mean FSH level was 30.3 ± 14.41 IU/L (reference threshold > 7.19 IU/L) [13], and the mean AMH level dropped to 31.7 ± 8.52 pmol/L (reference range: 13.9–151.38 pmol/L) [16, 18]. Overall, 34 of the 36 patients (94%) met criteria for Sertoli cell insufficiency. At the end of puberty, the mean inhibin B level is 17.3 ± 20.38 ng/mL far below the levels expected in a non‐KS adult (reference range 225–249 ng/mL).

Among the 36 patients who had completed puberty, 26 (72%) had initiated hormone replacement therapy (HRT), at a mean age of 16.6 ± 1.59 years.

### 4.4 Evolution of Leydig Cell Function During Puberty

A progressive increase in serum LH levels was observed during puberty, particularly within the first 2 years following pubertal onset (Figure 2A).

**Figure 2:**
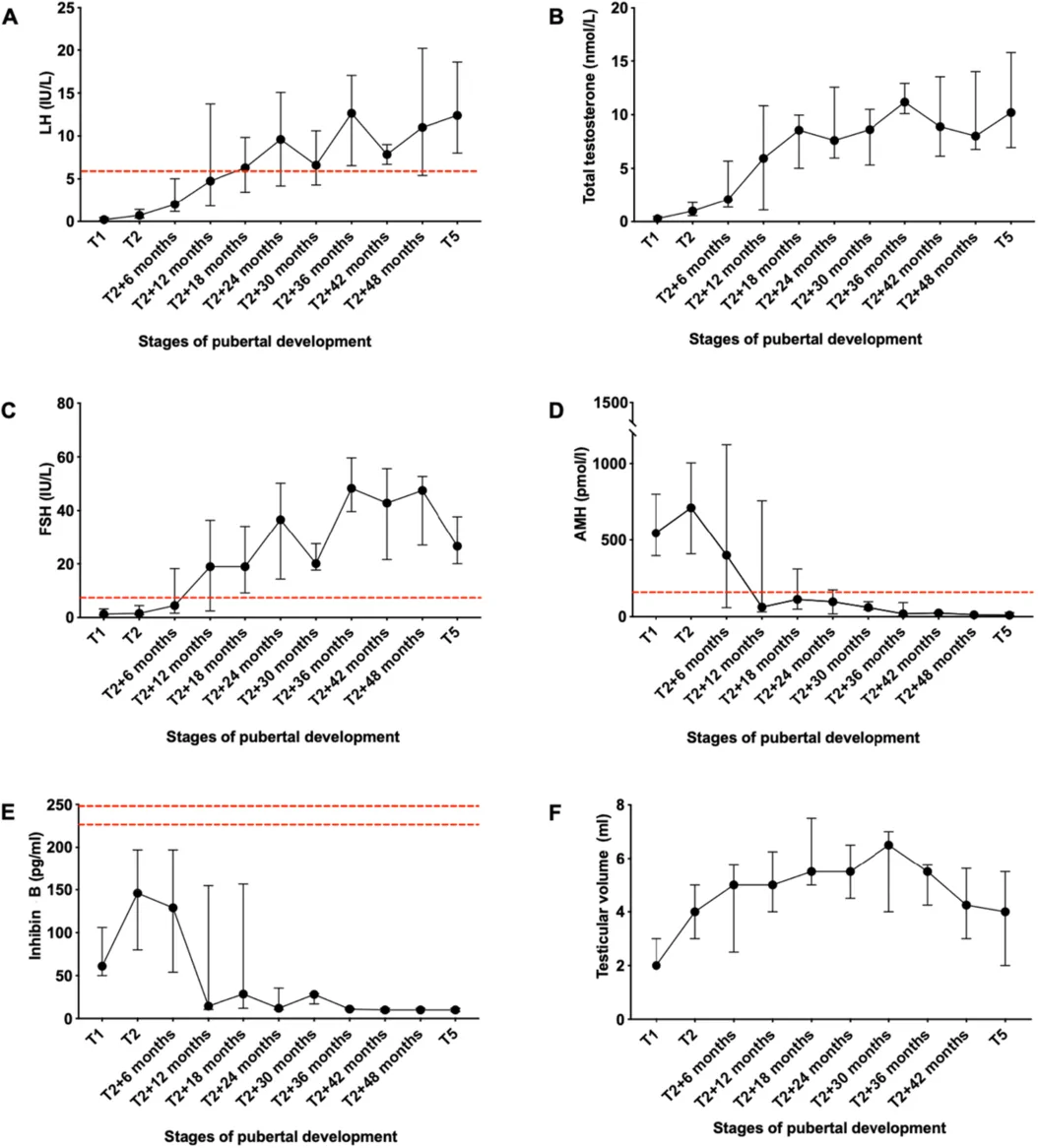
Kinetics of Leydig and Sertoli cell marker evolution during puberty. (A and B) Kinetics of Leydig cell marker evolution during puberty. (C–E) Kinetics of Sertoli cell marker evolution during puberty. (F) Kinetics of testicular volume evolution during puberty. The red dotted line represents the upper limit of normal serum levels for LH, FSH and AMH in (A), (C) and (D), respectively. In (E), the two red dotted lines represent the lower and upper limits of normal serum levels for inhibin B. In the graphs, black dots and horizontal bars indicate median values and interquartile ranges. Stages of pubertal development: T1 *Tanner 1*, T2 *Tanner 2*, T5 *Tanner 5*.

Serum testosterone levels increased steadily after pubertal onset, reaching a peak at approximately 18 months (Figure 2B). Thereafter, mean testosterone concentrations remained within the lower range of the expected values for Tanner stage 5 (reference range: 0.87–7.59 ng/mL; 3.0–26.3 nmol/L), consistent with a pattern of compensated Leydig cell dysfunction in a substantial proportion of patients.

### 4.5 Evolution of Sertoli Cell Function During Puberty

FSH levels increased rapidly following the onset of puberty and exceeded the Tanner stage 5 upper reference limit as early as 18 months after pubertal onset, indicating early‐onset Sertoli cell insufficiency in most patients (Figure 2C).

AMH levels declined markedly beginning at pubertal onset and fell below the lower reference threshold for Tanner stage 5 within the first year (Figure 2D). This pattern reflects early Sertoli cell maturation followed by progressive functional decline.

Inhibin B levels showed a transient increase between Tanner stages 1 and 2, but began to decline within 6 months of pubertal onset. Mean levels never reached the expected values for Tanner stage 5 (237 ± 12 pg/mL) and remained below the lower limit of normal for healthy adults (92 pg/mL) [19], reinforcing the evidence of impaired Sertoli cell activity throughout puberty (Figure 2E).

### 4.6 Evolution of Testicular Volume During Puberty

In this cohort, testicular volume increased at the onset of puberty and continued to rise over the following 30 months. After this point, a progressive decline in volume was observed. The maximum average testicular volume was reached approximately 30 months after pubertal onset, with a mean value of 6 mL (Figure 2F).

This volume is significantly lower than that observed in healthy boys at Tanner stage 3, where testicular volume typically reaches 10 mL, and it remains well below the expected volumes at Tanner stage 5 (approximately 20–25 mL). This trajectory suggests early testicular growth arrest followed by volume regression, which is consistent with progressive testicular failure.

### 4.7 Comparison of Phenotypes at the End of Puberty According to Diagnostic Circumstances (In Patients Not Receiving HRT)

Longitudinal hormonal kinetics were analysed in the entire cohort using time since pubertal onset.

The analysis of endocrine phenotype at puberty completion was restricted to patients who had clinically completed puberty at the time of data collection (*n* = 11). At the end of puberty, six boys with Klinefelter syndrome diagnosed prenatally and five diagnosed postnatally, who had undergone a complete hormonal evaluation at Tanner stage 5, were included in the analysis.

#### 4.7.1 Leydig Cell Function

At the end of puberty, a significant difference in LH levels was observed between the two groups. Patients diagnosed prenatally had a median LH level of 8.1 IU/L (IQR: 4.55–13.93), whereas those diagnosed postnatally had significantly higher levels, with a median of 16.6 IU/L (IQR: 15.2–21.8; *p* = 0.0303). In contrast, there were no significant differences between the groups in terms of total testosterone, bioavailable testosterone, or free testosterone levels (Figure 3).

**Figure 3:**
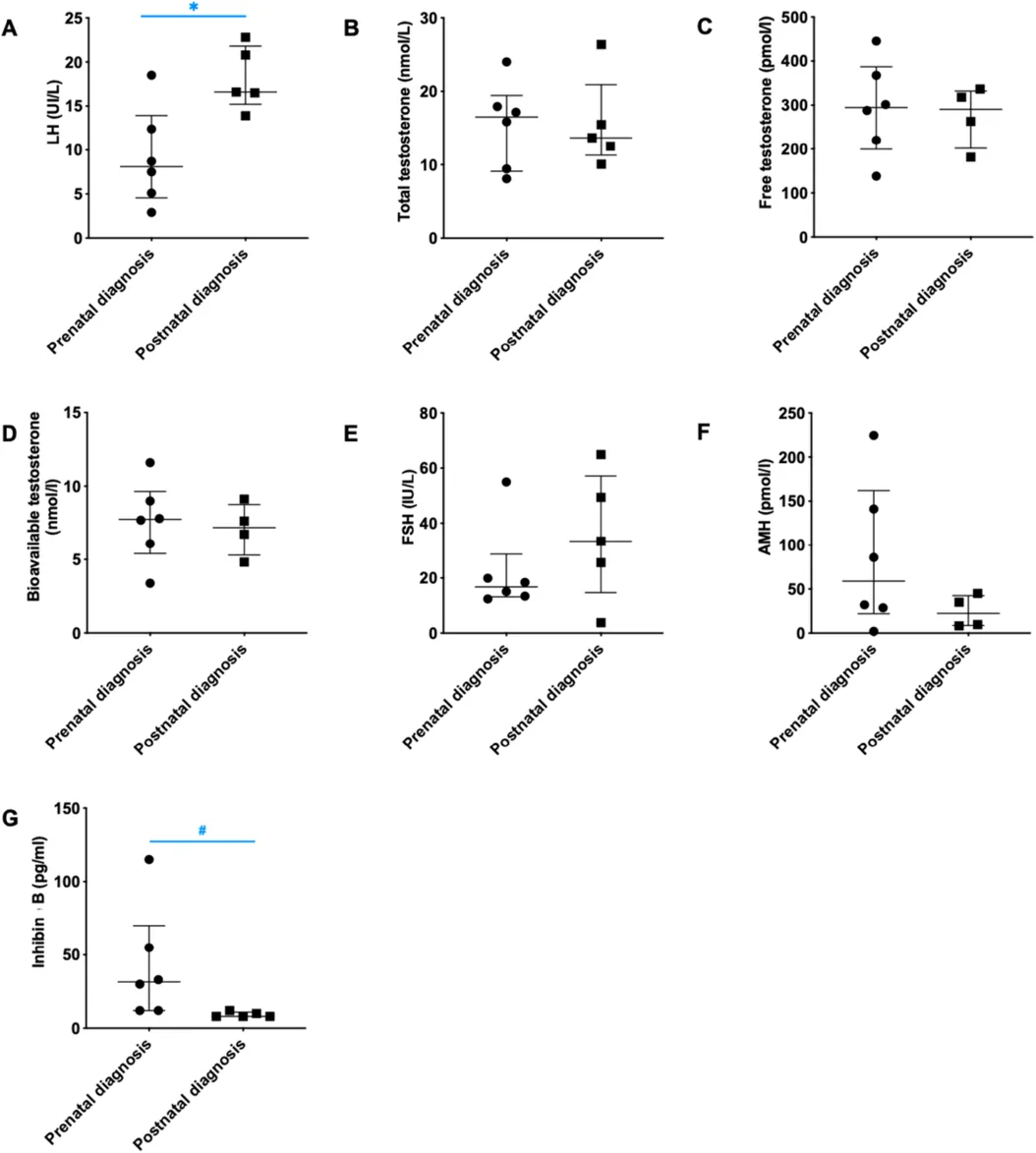
Comparison of Leydig and Sertoli cell function markers at the end of puberty according to diagnostic circumstances. At the end of puberty, six boys with Klinefelter syndrome diagnosed prenatally and five diagnosed postnatally, who had undergone a complete hormonal evaluation at Tanner stage 5, were included in the analysis. (A) comparison of serum LH levels (*p = 0.03). (B–D) Comparison of serum total testosterone (B), bioavailable testosterone (C) and free testosterone (D). (E–G) Comparison of serum FSH (E), AMH (F) and inhibine B (G, ^#^ *p* = 0.01). In the graphs, individual data points are represented by black dots or black squares. Horizontal bars indicate median values, and whiskers represent interquartile ranges. Statistical comparisons were performed using the two‐tailed Mann–Whitney *U* test.

#### 4.7.2 Sertoli Cell Function

Median FSH levels were 16.7 IU/L (IQR: 13.15–57.15) in the prenatally diagnosed group and 33.4 IU/L (IQR: 14.7–57.15) in the postnatally diagnosed group, although this difference was not statistically significant (*p* = 0.32) (Figure 3E).

Similarly, no significant difference was found in AMH levels between the two groups: 50 pmol/L (IQR: 22.03–162) in the antenatal group versus 22.5 pmol/L (IQR: 8.58–42.5) in the postnatal group (*p* = 0.47) (Figure 3F).

However, inhibin B levels were significantly lower in the postnatally diagnosed patients, with a median value of 8 pg/mL (IQR: 8–11), compared to 31.5 pg/mL (IQR: 12–70) in the antenatal group (*p* = 0.013) (Figure 3G). This suggests more pronounced Sertoli cell dysfunction at the end of puberty in patients diagnosed after birth.

## 5 Discussion

This study provides a comprehensive characterization of pubertal development and testicular function in a cohort of boys with KS who were diagnosed before or during childhood and followed longitudinally throughout puberty. To our knowledge, this is one of the largest single‐center studies describing the hormonal kinetics of Leydig and Sertoli cell function during pubertal progression in KS, using robust serial measurements. Our findings confirm the progressive nature of testicular failure in KS and highlight that both the timing of onset and the tempo of progression are influenced by the circumstances of diagnosis.

Consistent with previous reports, we observed that puberty generally begins spontaneously in boys with KS at a physiological age (12.6 years), with testicular enlargement and rising testosterone levels marking the initiation of pubertal development [10, 11]. However, the average duration of puberty in our cohort was 4.08 (±1.06) years, reflecting a slow pubertal tempo. This delayed progression is clinically relevant, as a pubertal duration exceeding 4 years is often considered a marker of incomplete or protracted puberty. The sluggish tempo likely reflects the progressive deterioration of testicular function, particularly Sertoli and Leydig cell insufficiency, which has been consistently reported in KS cohorts during adolescence [22, 23].

Our longitudinal hormonal data indicate that testicular function in individuals with Klinefelter syndrome begins to deteriorate early in the course of puberty. Notably, the decline in Sertoli cell function precedes that of Leydig cells. Within the first year following pubertal onset, we observed a rapid and sustained rise in serum FSH concentrations, alongside a marked decrease in AMH levels, indicating the early onset of seminiferous tubule dysfunction. Inhibin B levels followed a similar trajectory: after a transient increase between Tanner stages 1 and 2, levels declined progressively and failed to reach the normative range observed in healthy adolescents. By the end of puberty, the majority of patients exhibited inhibin B levels below the lower limit of adult reference values. These findings are in line with previous reports and further support the notion that Sertoli cell impairment is an early and predominant feature of testicular failure in KS, contributing to compromised spermatogenic potential from the initial stages of gonadal maturation [7, 8, 9]. Indeed, several studies have demonstrated that profound histological changes occur in the testes of individuals with Klinefelter syndrome around the time of hypothalamic–pituitary–gonadal (HPG) axis activation. As testosterone production begins to rise, there is a rapid acceleration of germ cell depletion, accompanied by progressive hyalinization of the seminiferous tubules, degeneration of Sertoli cells, and reactive Leydig cell hyperplasia. These processes are initiated early during puberty and appear to parallel the biochemical decline in Sertoli and Leydig cell function observed in our cohort [24]. Collectively, these findings suggest that the pubertal transition may act as a critical window during which irreversible testicular damage accelerates, reinforcing the importance of close monitoring during this developmental period.

Leydig cell function, assessed by serum LH and testosterone, showed a profile of compensated hypogonadism: testosterone increased during puberty but plateaued at the lower limit of Tanner stage 5 values, while LH rose progressively and exceeded the upper limit of normal about 3 years after pubertal onset. This hormonal pattern reflects a partial Leydig cell insufficiency, in which androgen production is maintained only through increased gonadotropic stimulation, primarily driven by hypothalamic–pituitary axis activation. Such a profile underscores the pituitary's compensatory response to declining Leydig cell capacity, a phenomenon that has been described in prior cross‐sectional studies of adolescents and young adults with KS [3, 4, 6]. Our study reinforces this model, suggesting that compensated hypogonadism in KS is not abrupt, but rather develops gradually throughout adolescence, becoming clinically significant by late puberty or early adulthood.

By the end of puberty, 80% of our patients exhibited biochemical evidence of Leydig cell insufficiency, and 94% demonstrated Sertoli cell dysfunction. These proportions are consistent with prior studies and reflect the nearly universal progression toward testicular failure in individuals with KS [6, 8, 25, 26, 27]. Our findings further support the concept that KS is characterised by early‐onset, progressive testicular insufficiency, with both the seminiferous and interstitial compartments affected during adolescence.

At the end of pubertal development, 72% of patients in our cohort were receiving testosterone replacement therapy (TRT), with a mean age at initiation of 16.6 years. According to the 2020 guidelines from the French Society of Endocrinology (SFE), TRT may be initiated from the age of 13 in cases of low testosterone levels and clinical signs of androgen deficiency. These recommendations advocate for a gradual titration of injectable testosterone doses to reach adult replacement levels by the end of puberty, when indicated [25, 26, 28, 29, 30, 31, 32]. However, the optimal timing and modality of TRT initiation in adolescents with KS remain subjects of ongoing debate.

One major consideration is the potential impact of exogenous testosterone on spermatogenesis. Several authors have raised concerns that initiating TRT prior to fertility evaluation may suppress endogenous gonadotropin secretion and thereby reduce the likelihood of retrieving viable sperm [11, 29, 30, 33, 34, 35, 36]. Consequently, some expert centres propose alternative strategies, such as combining testosterone with aromatase inhibitors to preserve gonadotropin‐driven intratesticular testosterone production until fertility preservation efforts have been undertaken [34]. Despite these evolving practices, there is currently no international consensus or formal guideline on the timing, dosing, or formulation of hormone replacement therapy in adolescent males with KS. These observations underscore the need for individualized, multidisciplinary management, integrating endocrine, reproductive, and psychosocial dimensions, and highlight the importance of prospective studies to better define the long‐term outcomes of different therapeutic strategies initiated during adolescence.

An important and novel finding from our study is the influence of diagnostic circumstances on the hormonal kinetics during puberty. To avoid confounding effects related to exogenous hormone administration, we deliberately excluded patients receiving testosterone replacement therapy from comparative analyses. Hormone replacement therapy (HRT) alters endogenous gonadotropin and gonadal hormone levels, and its inclusion could have obscured true physiological differences between groups. We thus compared two untreated subgroups: patients diagnosed prenatally, typically through incidental prenatal karyotyping, and those diagnosed postnatally based on clinical suspicion. This approach enabled us to demonstrate that postnatally diagnosed patients exhibited a more severe hormonal phenotype at the end of puberty. Specifically, LH concentrations reflecting Leydig cell stimulation were significantly higher in the postnatal group, suggesting more pronounced Leydig cell insufficiency. Similarly, inhibin B levels, an established marker of Sertoli cell function and spermatogenic potential, were significantly lower in postnatally diagnosed individuals. These findings suggest that the timing and circumstances of diagnosis may serve as proxies for underlying phenotypic severity.

To help explain the phenotypic differences observed between the prenatally and postnatally diagnosed groups, one plausible hypothesis relates to the timing and context of diagnosis. Prenatally diagnosed individuals were identified incidentally, most often in the setting of prenatal genetic screening unrelated to overt clinical suspicion, whereas postnatal diagnoses were made in response to medical signs prompting karyotype analysis. These clinical referral points, such as delayed puberty, gynaecomastia, or small testicular volume, may reflect more advanced or symptomatic forms of the syndrome. It is conceivable that individuals in the postnatal group would not have been diagnosed during childhood in the absence of overt clinical signs, given the known underdiagnosis of KS: only about 25% of affected individuals are identified during their lifetime [3, 26, 37]. The phenotypic variability among individuals with the same 47, XXY karyotype has been noted in multiple studies. As emphasised by Bonomi et al., most of what is known about KS phenotypes stems from clinically referred populations, which may overrepresent individuals with more severe or noticeable symptoms [1]. While genotype–phenotype correlations have not been clearly established in KS, and all patients in our cohort carried a homogeneous, non‐mosaic 47, XXY karyotype, the timing of diagnosis may nevertheless serve as a proxy for the severity of initial presentation.

Importantly, despite the milder hormonal profile observed in the prenatally diagnosed group, these patients still exhibited clear evidence of testicular dysfunction by the end of puberty. This observation underscores the value of early, pre‐symptomatic diagnosis: identifying KS before clinical signs emerge enables proactive monitoring and earlier intervention. Given the well‐documented long‐term consequences of untreated hypogonadism, including increased risk of metabolic syndrome, reduced bone mineral density, and cardiovascular morbidity [2, 6, 11, 26, 28, 30], timely diagnosis, even in individuals with a milder early phenotype, may play a crucial role in improving lifelong outcomes.

Finally, the strengths of our study include the homogeneous and longitudinally followed cohort, the availability of repeated standardized hormonal assessments, and the ability to compare diagnostic subgroups. However, several limitations should be acknowledged. The retrospective design may be subject to missing data and inherent selection bias. Importantly, prenatally diagnosed patients do not necessarily represent a fully unbiased reference subgroup, as prenatal karyotyping is typically performed in pregnancies with specific indications (e.g. advanced maternal age or abnormal screening results), which may introduce selection bias [2, 37]. The relatively limited number of patients in each subgroup may have underpowered some comparisons, particularly for testosterone measures. Additionally, semen analyses were not available, preventing direct correlation with fertility potential. Prospective studies combining hormonal profiling, semen evaluation and testicular imaging are needed to refine the predictive markers of spermatogenesis and to determine optimal windows for fertility preservation. Rather than stratifying patients by chronological age, we chose to assess hormonal dynamics based on pubertal progression, a more physiologically relevant approach given the wide age variability of pubertal onset. While Tanner stages 1 and 2 were well‐defined by testicular volume, intermediate stages were difficult to assign due to early‐onset atrophy. We therefore aligned hormonal data with months since the onset of puberty and used Tanner stage 5 norms as a consistent threshold to define Leydig and Sertoli cell insufficiency. This approach reduced the risk of overestimating dysfunction but may have underestimated its true onset in some patients. Intermediate Tanner staging was inconsistently documented and testicular volume rapidly plateaued or declined, limiting the reliability of defining transitions to Tanner stages 3–4. Therefore, hormonal kinetics were analysed according to time since pubertal onset rather than Tanner stage progression.

Future studies should examine the neurocognitive and psychological profiles of patients diagnosed prenatally versus postnatally, as differences in hormonal trajectories may reflect broader phenotypic divergence. Additionally, genomic and environmental modifiers influencing disease expression warrant further investigation. The development of standardized algorithms to guide fertility preservation timing, based on hormonal and developmental stage rather than arbitrary age cutoffs, represents a pressing clinical need.

In conclusion, this study confirms that testicular insufficiency in KS begins early in puberty, with Sertoli cell dysfunction preceding Leydig cell failure. The hormonal trajectory differs according to diagnostic context, with more severe phenotypes observed in postnatally diagnosed individuals. These findings underscore the value of early diagnosis and longitudinal endocrine surveillance, which may facilitate timely initiation of supportive therapies and optimise fertility preservation. Ultimately, personalized management strategies tailored to pubertal stage and diagnostic context may improve both short‐ and long‐term outcomes for adolescents with Klinefelter syndrome.

## Author Contributions

I confirm that all authors had access to the data and participated in the writing of the manuscript and have seen and approved the submitted version. T.A. contributed to the study design, execution, interpretation of data, manuscript draughting and critical discussion. C.L. and I.G. contributed to the study design, execution, interpretation of data, the inclusion of patients, manuscript draughting and critical discussion. S.B., C.C., F.M., A.‐L.B. and P.P. contributed to the study design, execution, analysis and interpretation of data, manuscript draughting and critical discussion. H.B. contributed to the inclusion of patients, study design, statistical analysis and interpretation of data, manuscript draughting and critical discussion. C.L. contributed to the inclusion of patients, study design, execution, analysis and interpretation of data, manuscript draughting and critical discussion.

## Funding

The authors have nothing to report.

## Conflicts of Interest

The authors declare no conflicts of interest.

## Data Availability

The data that support the findings of this study are available on request from the corresponding author. The data are not publicly available due to privacy or ethical restrictions.
